# Apples inside orange peels: Exploring the use of functional equivalents for comparing curriculum processes across contexts

**DOI:** 10.1177/17454999241258928

**Published:** 2024-06-12

**Authors:** Katherine M Caves, Ladina Rageth, Ursula Renold

**Affiliations:** 27219 ETH Zurich, Switzerland

**Keywords:** Comparative education, education operations, functional equivalence, education functions, education processes

## Abstract

Comparative education research is complicated by the difficulty of identifying comparable units across contexts. This paper considers the advantages and limitations of a functional equivalence approach to comparative education. The functional equivalence approach allows us to meaningfully compare the operations that serve each function in the full curriculum value chain of design, application, and updating. We use a theory-based list of common processes in each phase to develop a survey for experts from nine countries, then code their responses to derive ten key common functions. The functional equivalence approach allows us to aggregate some operations that serve the same functions, so our set of functional equivalents is slightly shorter than the theory-based list of processes. In comparing across contexts, we find easily identifiable functional equivalents, functional equivalents that manifest through very different operations, functional equivalents carried out by a wide variety of actors and institutions, similar operations that are not functionally equivalent, and functional equivalents that are not consistently present in all contexts. The functional equivalence approach helps identify comparable operations despite contextual diversity.

## Introduction

The challenge of comparing education programmes and systems has captured scholars’ interest for centuries, with its tension between identifying the variables that drive successful models and understanding the social factors of education (Hayhoe et al., 2017). Even early scholars warned against piecemeal copying and analysis that strips educational practices from their institutional foundations (Sadler, quoted in Bereday, 1964b). Simultaneously, comparative education has explored several methodological approaches that vary in how scientific, positivistic, intellectual, and institutional they are (Osborn, 2004; Välimaa and Nokkala, 2014; Manzon, 2018).

Comparing processes is a common approach but is challenging when processes are defined by socially constructed concepts (Brockmann et al., 2008, 2011). Therefore, a method that can identify equivalent elements by de-contextualizing and deconstructing that social construction is necessary. To address socially constructed concepts in comparative education, this study proposes analysing functionally equivalent operations. Unlike process comparison, functional equivalence compares elements that address similar functions (Park, 2011; Teichler, 2014). Based on theoretical reasoning and substantive knowledge, this approach starts by defining specific functions (‘Bezugsgesichtspunkte’, Luhmann, [1970] 1991: 17), from which dissimilar operations can be compared via functional equivalence (Luhmann, [1970] 1991).

In this paper, we explore the advantages and possibilities of comparing education programmes via functional equivalents. Specifically, our research question is: **what are the advantages and limitations of a functional equivalence approach to comparative education?** Although functional equivalence is an established concept, it is not widely used in comparative education. A key reason for this is the gap between Luhmann’s theorized functional approach (Luhmann, 1964) and a practical comparative method for education and training programmes (Schriewer, 1987, 2021; Zolo, 1986).

Luhmann ([1970] 1991) recommends starting from a pre-defined set of functions – which serve as ‘regulative schema’ (Schriewer, 2000: 40f.) – and a range of operations that can be used as reference points to identify functional equivalents, or operations that serve the same function ‘despite being incomparably different as concrete events’ (*ibid*, p.17). We use the term ‘operations’ consistently throughout this paper to refer to Luhmann’s ([1970] 1991) original German *Leistungen*, which is often translated to ‘performances’. If those operations solve the same fundamental problems or serve the same function, they become functional equivalents. Schriewer (1987) refers to these operations as ‘the production of effects’, ‘input and output performances’, ‘performances’ (p.37), ‘systems strategies’, or ‘problem solutions’ (p.41).

We explore this approach by identifying functional equivalents across education programmes: First, we list the processes of education programmes described in existing theory and literature and organize them into curriculum design, application, and updating phases (e.g. Marsh & Willis, [1984] 1995; Kelly, [1977] 2009; Billett, 2006, 2011). Second, we gather data on the operations and actors behind those processes in education and training programmes in diverse contexts via an expert survey, and code their responses using qualitative content analysis (e.g. Mayring, 2004, 2014; Schreier, 2014). Third, we identify the functions and derive the functional equivalents across all programmes, enabling us to look through socially constructed concepts. Lastly, we consider how this equivalent-function-based comparison performs. We find that our functional equivalents generally align with the processes identified in the literature. However, applying functional equivalence as an empirical approach generates broader functions than what we expect based on the processes described in the literature. We note that the programmes we study – although of the same type and level – perform each function through different operations and with various actors and institutional settings. This finding indicates that a functional equivalence approach performs well when there are different solutions to similar problems. Functional equivalence can help identify comparable operations despite different social, economic, and institutional contexts. Comparing those elements can uncover variation in the ways that different programmes address similar challenges and facilitate further comparative research.

## Literature review

### Challenges and approaches of comparative education

Modern methods have made it possible to compare swaths of data, yielding important insights. However, some comparisons risk naïve empiricism and – at worst – have been criticized for limited development relevance or colonialist perspectives (Hayhoe et al., 2017; Komatsu, 2017). In education sectors and types with extensive variation across contexts, comparison can be misleading if it ignores different operations serving the same function or compares similar operations that serve different functions. This challenge is especially relevant for comparing education and training programmes due to their high diversity and variety of involved institutions from education and employment systems (Billett, 2011).

A relatively large body of research compares similar education systems and programmes, often focussing on similar institutions (e.g. Allmendinger, 1989; Kerckhoff, 1995; Ryan, 2000, 2001; Streeck, 1989; Streeck et al., 1987). In policy research, similar institutions are the accepted starting point for cross-country analyses. For example, the OECD often uses an institutional framework (e.g. OECD, 2015).

Economists and sociologists often compare education and training programmes and systems that solve the same fundamental problems – for example, programmes intended to reduce unemployment or raise literacy. Each discipline takes its own approach to identifying fundamental problems and functionally equivalent operations.

The economic literature frames fundamental problems as market failures, highlighting how different institutions affect efficiency and resolve issues around information and incentives (e.g. Eichhorst et al., 2015; Wolter and Ryan, 2011). Using these fundamental problems, researchers like Escandon-Barbosa et al., emphasize that different institutions can provide ‘other channels by means of which a country’s institutional weaknesses may be counteracted’ (2019, 14).

In sociology, institutions are a widely used starting point for comparative analysis. In the 1980s and 1990s, the comparative stratification literature proposed institutional dimensions along which national education systems differ (e.g. Kerckhoff, 1995). These dimensions are the degree of standardization in educational curricula, the level of stratification (extent and form of tracking), and the vocational specificity of education and training. Rageth and Renold (2020) provide a theoretical framework for comparing vocational education and training programmes across countries and cultures based on the institutionalized relationships across the education and employment systems.

Names, terms, and concepts are socially constructed, so cross-contextual or international comparisons risk analysing non-comparable terms and concepts (Brockmann et al., 2008, 2011). According to Grollmann (2008), scholars may apply their own culturally determined terms and concepts, thereby introducing bias from their own cultural projections (‘nostrification’; Grollmann, 2008, 256ff). Renold (2020) affirms the importance of decontextualizing and deconstructing social constructs in comparative education research.

Scholars of comparative education take various approaches to addressing the challenge of comparability given socially and culturally constructed concepts. For example, Brockmann et al. (2008, 2011) recommend using transnational categories to avoid false comparisons of outwardly similar terms and concepts with culturally distinct meanings (Brockmann et al., 2008, 2011). In addition, Kauko and Wermke (2018) recommend a contingency-based approach to comparative education that takes path dependency and development over time into account.

Approaches that draw on sociology and anthropology try to avoid social-construct bias by situating educational practices in traditions, institutions, and history, but these approaches can be too specific and detailed for broader interpretation or multi-context comparison (Hayhoe et al., 2017). In contrast, methods like the famous four steps of comparison (description, interpretation, juxtaposition, and comparison; Bereday, 1964a; Hilker, 1962) are a useful starting point for comparing across small or medium sets, but are criticized for lacking structure (Adick, 2018). Dealing with socially and culturally constructed concepts remains a challenge in comparative research.

According to Grollmann (2008), another challenge of comparative education research is finding the appropriate level or unit of analysis – the balance between too much complexity and too much simplicity. Lauterbach and Mitter (1998) argue that the analytical level must account for comparability. The most common unit of analysis in comparative education is the nation-state, with considerable discussion on the need for both smaller and larger units, or simply more thoughtful units (Kim, 2017; Wolhuter, 2008). Torres et al. (2022) link unit of analysis with method, highlighting, for example, how ethnography tends to have smaller units of analysis than methods that aggregate population-level data quantitatively. The consequences of the wrong unit of analysis can be significant – an example from the classroom discourse literature shows how using the wrong unit can lead to misleading results (Lefstein et al., 2015). Taken together, the challenge of comparability is not just an issue of names and concepts but also one of finding a similar unit of analysis.

### Functional equivalence

Luhmann (1964, 2012–2013, [1970] 1991) articulated functional equivalents as a means of comparison that addresses both the issue of names and concepts and the issue of unit of analysis. He argued that, although the specific solutions to social problems may vary, all solutions serving the same function are comparable. Identifying operations related to functionally similar problems and comparing only those operations makes comparisons relational rather than similarity-based (Luhmann, 2010). Berry (1969) used the concept in ethnography, describing functional equivalences as social behaviours that ‘developed in response to a problem shared by two or more social/cultural groups, even though the behaviour in one society does not appear to be related to its counterpart in another society’ (p.122). These approaches are based on the central insight that ‘different elements can respond to the same problem’ (Michaels, 2006: 56).

A few scholars of comparative education have taken up functional equivalents. Välimaa and Nokkala (2014) differentiate between lexical and ‘effective’ or functional equivalence for comparative higher education research, stating in practical terms, ‘Instead of aiming to find the actors with the same name in different systems of higher education we should try to find higher education actors which serve the same purpose in different systems of higher education’ (p.426). Teichler (2014) also applies functional equivalents to comparative education, defining them as ‘different mechanisms serve the same purposes (or, in reverse, how identical mechanisms serve different purposes from country to country)’ (p.397). However, these studies do not focus on the potential of applying a functional-equivalents approach to comparative education research.

Schriewer (2021) explores the development of comparative research from a theoretical perspective. In his discussion of Luhmann’s (2012–2013) functional approach to comparison, he expresses surprise that the method has not been taken up more in comparative education, stating ‘the extensive ignoring in Comparative Education of the theoretical tools conveyed by the work of Niklas Luhmann is surprising given that his “functional” method is, at its core, a comparative method’ (Schriewer, 2021: 447). Earlier, he argued for the potential of Luhmann’s approach to revitalize comparative research and its utility for structuring comparative research in various areas of education (Schriewer, 2000). Schriewer (2021) attributes the underutilization of the functional equivalence and similar approaches to its relatively opaque theory and abstractness. Wider application of functional equivalence in comparative education requires some translation to the concrete terms commonly used to describe methods, which must be accomplished without oversimplifying complexity, temporality, or contingency.

Moreover, Zolo (1986) analyses the epistemological ramifications of Luhmann’s functional equivalence approach and finds serious limitations in its decision to reduce complexity for comparability, while acknowledging its practical benefits. We refrain from a causal understanding of functionalism, instead using it as a method (Luhmann, 1964, [1970] 1991) or a ‘specific observation attitude’ (Schriewer, 2000: 37), which helps us organize complexity in a meaningful framework – regarding the field and question under analysis – and to identify comparable units (Zolo, 1986). In addition, as functions signify the meaningful framework for the analysis of functional equivalents, this approach depends heavily on the definition of the functions, which – according to Luhmann ([1970] 1991) – should happen based on theory and substantive knowledge. However, such an approach may ignore those operations that may serve non-obvious functions that are difficult to observe. Moreover, functionalism is often criticized for its failure to account for social change and individual agency. Nevertheless, based on the literature we argue that functional equivalence can solve practical problems as a method, and therefore limit our consideration of it to its methodological applications.

## Methodological approach

To address our research question about the advantages and limitations of a functional equivalence approach to comparative education, we explore the potential of a functional equivalence approach in comparative education by applying it to a comparison of the main curriculum operations in similar education programmes across diverse contexts. This empirical case is an example of how a functional-equivalents approach might be used in a comparative education setting. It enables us to explore the utility of the functional-equivalents approach concretely.

The start of Luhmann’s (1964) functional method is constructing a ‘comparative range of equivalent operations…from which different possibilities can be understood from a uniform perspective’ (Authors’ translation of Luhmann, [1970] 1991: 14). These operations then become reference points from which ‘individual activities then appear as equivalent, interchangeable, fungible, despite being incomparably different as concrete events’ (*ibid*, p.17). This process enables the researcher to identify different solutions to the same social problems and assess their functional equivalence (Braun, 2006; Dogan and Pelassy, 1984; Luhmann, 1964, 2010; Schriewer, 1987, 2000). Functional equivalents can then be compared.

Therefore, we begin by defining a unit of analysis – in our case education programmes in the same level and type of education (see section on unit of analysis). Next, following Luhmann (1964), we construct our comparative range of operations – these will become the reference group to identify our potential functional equivalents. We define a framework to structure our range of operations based on the literature (see section on deductive approach). We then populate the framework both inductively and deductively – deductively by deriving the processes identified in previous literature (see section 3.3), and inductively by conducting an expert survey to gather data on operations (see section 3.4). We combine the two sources using qualitative content analysis, which enables us to test the application of a functional equivalence approach to comparative education and discuss its advantages and limitations.

### Unit of analysis

Comparison at the programme level is not unusual (e.g. Rageth et al. (2021), but it is complicated by the issue of finding similar programmes. Luhmann ([1987] 2009) argues that education systems prepare young people for society, building human capital and assigning social positions through selection. However, individual programmes may serve different purposes, and therefore emphasize different functions.

Education programmes are defined by their level (ISCED^1^) and type. Education systems are typically general through compulsory education, then starting at the secondary or tertiary level (ISCED level 3+), most systems offer programmes in multiple types like academic and vocational/professional (UNESCO, 2012). For example, the academic education type typically includes academic secondary education then bachelor’s, master’s, and doctoral degrees at universities. The vocational/professional type typically includes vocational education and training (VET, sometimes called technical and vocational education and training, TVET) at the secondary level and various levels of professional education and training (PET, sometimes called higher TVET) at ISCED levels 5+ (UNESCO, 2012). Often there is only one programme within a given level and type, but there may also be multiple programmes (e.g. bachelor of science and bachelor of arts). Focussing on the programme level reduces complexity and gives us comparable units – programmes at the same level and type – while allowing for the diverse organizational characteristics that determine the operations of different education programmes.

We focus on upper-secondary-level VET programmes that combine education and training. Importantly, these programmes prepare young people not only for higher and further education but also for professional life on the labour market. Thus, they operate at the intersection of the education and employment systems. The need for communication and coordination between actors from the education and employment systems is the constitutive element of education and training programmes, distinguishing them from other education programmes (Rageth et al., 2021). The specificity of these programmes is particularly interesting for our purpose because Luhmann’s approach draws on systems theory and the issue of social communication (Schriewer, 2021).

### Deductive approach: drawing on the literature

Luhmann (1964) recommends applying theory to define the comparative range of functions. However, the literature generally does not take a functional approach and we do not have the luxury of deriving all functions involved in education programmes from theory. Instead, the literature typically refers to educational ‘processes’. Therefore, we add a step. Luhmann (1964) recommends proceeding directly from theoretically derived functions to empirically observed operations that we can aggregate by function into functional equivalents. Instead, we analyse the literature to deductively derive the processes identified in education programmes. We define a comparative range not of functions but of processes. That comparative range then structures the identification of functional equivalents once we collect empirical operations (see section on inductive approach).

Many researchers have articulated the idea of curriculum as a process rather than solely as a content that an education programme needs to deliver (e.g. Marsh & Willis, [1984] 1995; Billett, 2006, 2011; Kelly, [1977] 2009). This wider perspective allows researchers to consider power structures and control activities (Marsh & Willis, [1984] 1995). Renold et al. (2015) apply the idea of curriculum as a process to a three-phase cycle, calling it the curriculum value chain (CVC). We use the CVC as a structuring principle, helping us consider the full range of functions involved in an education programme and balance complexity and simplicity (Braun, 2006; Grollmann, 2008).

**Figure 1.**
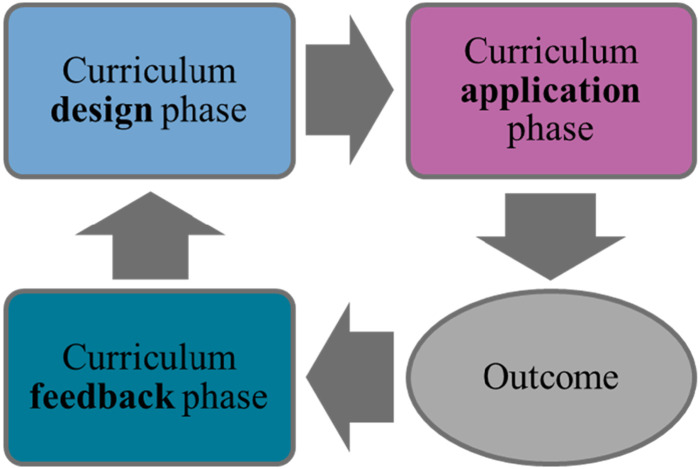
The curriculum value chain (CVC). Source: Renold et al. (2015: 13).

Figure 1 shows that the CVC is a cycle with three phases in which operations are observable. First, in the *curriculum design phase*, actors define and decide upon the ‘intended’ (Billett, 2006, 2011) or ‘planned’ (Marsh & Willis, [1984] 1995; Kelly, [1977] 2009) curriculum, thus specifying curriculum content, qualification standards, and examination forms.

Second, the *curriculum application phase* covers programme delivery, that is, who is taught, by whom, where, with what equipment, and financed by whom. Billett (2006) builds on Marsh and Willis ([1984] 1995) to differentiate between the enacted curriculum and the experienced curriculum, and Kelly ([1977] 2009) makes a similar distinction between delivered and received curricula.

The outcomes following the curriculum application phase show whether the curriculum had its intended effects (Finch and Crunkilton, 1993). In the *curriculum feedback phase*, this information is gathered to help determine curriculum update content and timing, which re-start the cycle (Renold et al., 2015).

In the curriculum design phase, the *instalment of a reform group* is the first process often mentioned in the literature (Robinson, 1981; Tyler, 2013; Vollstaedt, 2003). Next are *the process and method for curriculum development* (Kelly, [1977] 2009; Eggenberger et al., 2018) and *definition of the curriculum content and orientation* (e.g. Marsh & Willis, [1984] 1995; Billett, 2006, 2011; Kelly, [1977] 2009). The literature mentions two additional processes related to curriculum design: *legitimation of the curriculum* (Vollstaedt, 2003) and the *decision-making process* (Finch and Crunkilton, 1993).

In the curriculum application phase, we find six processes in the literature: *career guidance and counselling* (Billett, 2011), *student enrolment* (Billett, 2006, 2011), *qualification of personnel* (Billett, 2006, 2011; Tyler, 2013), *provision of learning and training material* (e.g. Finch and Crunkilton, 1993; Marsh & Willis, [1984] 1995); Billett, 2006, 2011), *programme delivery* (e.g. Finch and Crunkilton, 1993; Rageth and Renold, 2020), and *programme output measurement* (Billett, 2011; Kelly, [1977] 2009).

In the feedback phase, the literature mentions four processes: *determination of responsible actors* (Kelly, [1977] 2009), the *information-gathering process* (e.g. Billett, 2011; Finch and Crunkilton, 1993; Tyler, 2013), *update timing decision process* (Rageth and Renold, 2020), and *launching a reform process* (Billett, 2011). The following table summarizes these processes:

We use the processes in Table 1 as a theory-driven codebook for qualitative content analysis of the expert survey to structure the empirically derived operations (for the detailed codebook including sub-level processes, see part A of the online supplemental material). Qualitative content analysis helps scholars systematically analyse textual data (Mayring, 2004, 2014; Schreier, 2014). Mayring (2004) describes the process: break text (data) into single analytical units and examine those based on a system of categories or codes. Codes tag data with units of meaning (Miles and Huberman (1994) and the codebook or coding frame structures data analysis (Saldaña, 2009; DeCuir-Gunby et al., 2011; Schreier, 2012). Detailed codes specify sub-level processes and capture information about main codes (Saldaña, 2009; Schreier, 2012). For example, the respondents may mention a specific curriculum development method, making it a sub-code under the code ‘process and method for curriculum development’.

**Table 1.** Range of processes deductively derived from the literature.

Curriculum design (1.00)	Curriculum application (2.00)	Curriculum feedback (3.00)
1.10 Instalment of a reform group	2.10 Career guidance and counselling	3.10 Determination of responsible actors
1.20 Process and method for curriculum development	2.20 Student enrolment	3.20 Information-gathering process
1.30 Definition of the curriculum content and orientation	2.30 Qualification of personnel	3.30 Up-date timing decision process
1.40 Legitimation of the curriculum	2.40 Provision of learning and training material	3.40 Launching a reform process
1.50 Decision-making process	2.50 Programme delivery	
	2.60 Programme output measurement	

Codebook development is an iterative process (Saldaña, 2009; DeCuir-Gunby et al., 2011). We were conscious that specifying codes includes making choices about analytical focus, and we remained open to new codes during analysis (Schreier, 2012). We developed theory-driven codes and revised them based on trial coding of a pre-test among selected experts.

### Inductive approach: expert survey

Next, we turn from the theoretically derived processes to empirically derived operations. In a qualitative online survey, we asked about the main operations in each CVC phase of the largest upper-secondary education programme preparing students for labour market entry in respondents’ countries or states.

The survey was structured based on the CVC, so respondents were asked to list the main operations for developing, applying, and updating the curriculum (for the detailed questionnaire, see part B of the online supplemental material). We defined main operations as the tasks that actors must fulfil until, for example, in the design phase, the intended curriculum is developed. The survey asked for operation names, brief descriptions, and the involved actors. This information is the only data included in the qualitative content analysis.

We pre-tested the survey and codebook among four selected experts and revised the two. In the survey revision, we asked for at least one operation each before, during, and after defining the curriculum, applying the curriculum, and evaluating the curriculum. Moreover, as delineating between the curriculum updating and the curriculum development was not straightforward, we clarified that updating is about gathering information and deciding when to update, not actually updating content. In the codebook revision, two researchers coded pre-tests independently, then compared their codes and discussed the mismatching ones (Krippendorff, 2004; Mayring, 2004, 2014). We added and re-ordered a few sub-level codes based on the pre-test answers and renamed some processes codes to better encompass sub-level processes. Table 2 summarises the revised processes:

**Table 2.** Revised range of processes after pre-test.

Curriculum Design (1.00)	Curriculum Application (2.00)	Curriculum Feedback (3.00)	Actors
1.10 Curriculum development	2.10 Career guidance and counselling	3.10 information-gathering process	A Teacher
1.20 Curriculum legitimation	2.20 Student enrolment	3.20 Up-date-timing decision process	B Company
1.30 Curriculum approval	2.30 Qualification of personnel		C Professional association
	2.40 Provision of syllabus, material, and infrastructure		D Ministry of Education
	2.50 Programme delivery		E Ministry of Labour
	2.60 Programme output measurement		F Policy maker
			G Pedagogical institute/teacher university
			H Researcher
			I Principal/school
			J Ministry of education affiliated agency
			K Other

The survey sample includes 30 participants in a professional development programme for international education leaders. Convenience sampling of programme participants enabled us to cover different countries at all development stages and on various continents.

We performed qualitative content analysis at the programme level as unit of analysis. We first aggregated the data from different respondents on the same programme and then coded this aggregated data. This approach helped us clearly see the main operations for each programme. Most respondents answered in English, and we translated three Spanish-language responses before coding.

To ensure objectivity, two coders independently coded programme-level responses then compared codes (Krippendorff, 2004; Mayring, 2004, 2014). Agreement percentage (Feng, 2014; Mayring, 2014; Schreier, 2012) is 67.5% for the main operations (not sub-level codes). However, agreement varies by question and respondent. Most coding disagreement comes from curriculum design- and feedback-phase overlap, so discussion helped sharpen this area.

### Deriving functional equivalents

As Luhmanns approach remains too abstract for application, we follow Renold’s (2020) more practical procedure for deriving functionally equivalent operations across education and training programmes. This approach builds on DiFrisco, who suggests to use operation-name analysis (‘treatment’) and descriptions (‘conditions’). He defines functional equivalents as distinct traits that have the same function: ‘Trait T has the function F in country x under conditions Cn if T’s performing F in x under Cn contributes to attaining the goal G’ (2017, 2). Decontextualizing the treatments or traits (operations) in education programmes (cases) and structuring them by the processes identified in the literature, helps us identify the operations’ common functions based on their conditions and goals. From these functions, we then derive functional equivalents from the main operations identified by our respondents.

## Results

This section presents results of our empirical case, which we use as an example to explore the advantages and limitations of functional equivalents as a comparative approach. Our 30 survey respondents are high-level leaders in education governance, school leadership, research, industry associations, and NGOs. As mentioned in the section on our inductive approach, respondents are drawn from a professional development programme for education leaders. Participants come from twelve education and training programmes at the national or state level in nine countries. Countries represent high-, middle-, and low-income countries and are spread across Africa, Asia, Europe, Central America, North America, and South America. Respondents’ answer quality varies, but due to their participation in the professional development programme and in-depth discussion of programmes with coders and other researchers, we were able to determine when answers were clearly wrong, such as incorrect levels or programme orientations.

**Figure 2.**
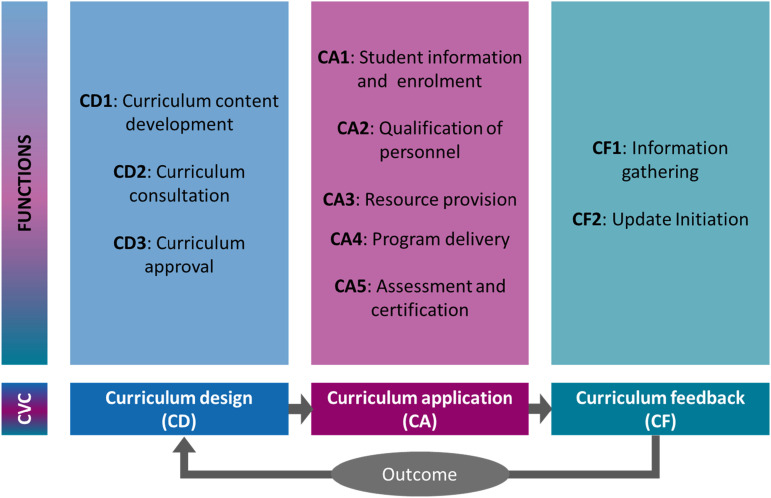
Functions in education and training programmes.

Even with practical challenges, applying a systematic procedure helped us identify functional equivalents and main functions at the programme level. This speaks to the feasibility of the functional-equivalents approach. After coding each operation based on the process codes, we considered the operation’s function independent of context to derive the functional equivalents. The resulting ten functions – three in curriculum design, five in curriculum application, and two in curriculum feedback – are closely aligned with the processes identified in the literature (see section on deductive approach; e.g. Billett, 2011; Kelly, [1977] 2009). These functions are theoretically decontextualized from local institutional and actor configurations, so we can meaningfully compare the operations serving those functions across contexts. Figure 2 shows the functions.

Detailed information about how we derived the functional equivalents and about each function in each context is shown in part C of the online supplemental material. The following subsections use specific examples to illustrate some advantages and challenges we encountered in using a functional equivalence approach. Specifically, we highlight examples of easily identifiable functional equivalents, situations where very different operations aligned with the same functional equivalent, situations where very diverse actors or institutions are involved in the same functional equivalent, and similar-seeming operations that belong to different functional equivalents. We also show examples of functional equivalents that are not consistently found in all contexts.

### Easily identifiable functional equivalents

Many functional equivalents were relatively easy to combine and had high inter-coder consistency because the operations solving the same fundamental problem were recognizably similar. These are examples where the functional-equivalents approach finds comparable units that might be observable without such careful decontextualization. For example, every analysed programme includes the function of developing curriculum content, thus operations serving this function can be identified as functional equivalents. The curriculum content itself is variously called the qualification profile (case 12), qualification standard (cases 2, 3, 4), qualification (case 10), state standards (case 9), occupational skills profile (case 5), or competencies (cases 3, 7, 8). However, although the operations refer to curriculum content by different names, these different terms are all clearly related.

Another example is update-related information gathering (CF1). Most cases mention ‘monitoring’ or ‘evaluation’ and describe gathering data on programme impact and curriculum relevance. Some cases also include information on students’ labour market outcomes. These examples are straightforward not only from a functional equivalence approach but also with other methods.

### Functional equivalents with diverse operations

Education and training programmes provide fertile ground for situations where different operations fit into the same functional equivalent. These programmes tend to have diverse operations and institutions, and can have different learning places (Billett, 2011). Depending on the programme, the CVC can cover the curriculum for school, the workplace, or the two. These functional equivalents would be difficult to identify with another approach.

The function of programme delivery (CA4) comprises teaching and training students, thus all operations of school teaching, workplace training, or similar. All cases fulfil this function with at least one operation, however, the social constructs for these operations vary greatly – respondents used terms like ‘execution’, ‘training process’, ‘delivery of content’ or ‘implementation’. Fully school-based programmes summarized this operation with phrases like ‘Teachers deliver instruction’. In contrast, programmes with work-based learning would include other learning locations, for example, ‘Deliver curriculum for workplace, school and intercompany courses’. All operations in this functional equivalent serve to deliver the curriculum to students, but the concrete operation and socially constructed concepts used to describe it can be very different.

### Functional equivalents carried out by diverse actors

The functional equivalent approach also enabled straightforward aggregation in cases when very different actors are involved in functionally equivalent operations. Again, these would be difficult without taking a functional-equivalents approach. Education and training programmes differ in their degree of linkage between the education and employment systems (Rageth and Renold, 2020), meaning different actors may have power over similar operations in different programmes.

For example, most cases mention operations related to the function of personnel qualification (CA2). The corresponding functional equivalent entails selecting and preparing personnel for programme delivery (‘capacity building’, ‘professional development’), but personnel can include professionals, specialists, teachers, and trainers. These personnel may work in schools, training centres, or training companies. The examples below show the actors carrying out operations in this functional equivalent for a few selected cases:

• Case 2: Teacher/pedagogical university and schools/principals
• Case 3: Ministry of Education and Ministry of Labor
• Case 4: Vocational training institute
• Case 6: Schools/principals and teachers
• Case 7: Ministry of education agency, teachers, and professional associations
• Case 8: Companies.

Without a functional equivalence perspective, it may be difficult to identify operations carried out by such different actors that are comparable because they serve the same function.

### Similar operations that are not functionally equivalent

As predicted by Teichler (2014), we find some examples of operations that have similar names or socially constructed concepts yet are not part of the same functional equivalent. For example, Case 12 refers to ‘development of an education plan’ in curriculum content development (CD1), and Case 10 refers to ‘teachers develop lesson plans’ in resource provision (CA3). The word ‘plan’ here means very different things – one is a framework curriculum of competencies, and the other is a guideline for delivering a specific lesson. The de-contextualization of these operations and focus on the functions that they serve help us avoid confusion here.

### Functional equivalents not present in all contexts

All the above situations – clear equivalence, different operations, or actors in one functional equivalent, and multiple functional equivalents related to apparently similar operations – were predicted in the theory on functional equivalence. We encountered another situation that is not as clearly indicated in the theory. Luhmann does not argue that every function must appear in every context, but he does note that social systems with similar purposes converge to carrying out similar functions. We find some functional equivalents that do not appear in all contexts.

Two codes in student information and enrolment (CA1) demonstrate two potential interpretations for this missingness. On one hand, a missing function can indicate a weakness of the programme. For example, few cases reported operations related to career guidance and counselling. One case mentions student information sessions and one mentions career guidance and materials. This missingness most likely indicates a real lack of career guidance and therefore a programme weakness because the programmes without career guidance and counselling are missing part of their function.

In contrast, respondents rarely mentioned operations related to student enrolment. These operations are mostly called student selection and enrolment, although one case mentions defining a target enrolment strategy and using programme marketing to attract students. Another case described contracting companies for work-based learning. In this case, we have reason to be sceptical of the data. All the programmes have students, so they must have some operation related to enrolment. Because of our strong background knowledge on each case, the interpretation here is not programme weakness but missing data – participants either forgot or neglected to include this operation in the curriculum application phase.

## Discussion

Our focus is on the application of a functional equivalence approach to comparative education – not the specific findings of our survey – and its advantages and limitations. In this section, we explain two advantages and two limitations – after reflecting on the analysis carried out in this paper – of functional equivalence as a method for empirical comparative research.

First, we find that the functional equivalence approach is not more difficult than other comparative approaches and is adaptable to use with various data sources and empirical methods. We acknowledge that it can be a challenge to understand what the method should be from Luhmann’s theory-focused work, but there is additional literature reflecting on how we can practically apply the equivalent functions approach. We extracted a concrete process – define the unit of analysis, use theory and literature to structure the range of comparable processes, empirically collect concrete operations related to those processes in the units under study, and decontextualise and deconstruct the operations to derive the common functions and identify functional equivalents. Our application of this process was straightforward, and we found not only the easily identifiable functional equivalents but also functional equivalents that we did not expect from the previous literature.

Second, we find that a functional equivalence approach makes it possible to uncover and compare operations that may be misclassified or ignored otherwise. In our analysis, we found functional equivalents where the concrete operations and actors were diverse, as well as operations with similar socially constructed names that fit into different functional equivalents. The actor variation we found in our analysis reinforces the goal of identifying diverse solutions to shared problems and indicates that the functional equivalence approach has enabled us to compare similar elements despite major institutional differences. Searching for similar institutions across contexts – for example, career guidance and counselling offices – would make it more difficult to observe the many ways that student information and enrolment is achieved across diverse programmes. This advantage is an indication that the exciting potential of a functional equivalence approach can work in a real empirical setting.

Third, we find that functional equivalence does not reduce the need for deep understanding of the phenomena and units under study. This is not a comparative method that facilitates very large-scope empirical work. In contrast, constructing the range of comparable operations requires a careful study of existing theory and literature. In addition, interpretation of empirically derived operations requires at least moderate understanding of the units under study. In our case, certain coding tasks would have been impossible without our knowledge of each programme. Perhaps other approaches to collecting empirical operations could facilitate less-content-intensive ways of coding operations into functional equivalents. Regardless of method, though, context knowledge is required for interpretation of findings, for example, why some functional equivalents did not appear in every unit under comparison.

Fourth, we find that the utility of the method is constrained by the quality of literature related to operations and functions under study, especially if the literature guides the derivation of the pre-defined set of functions – as suggested by Luhmann ([1970] 1991) – and thus the ‘regulative schema’. In our case, curriculum content development is well covered by the literature, while curriculum validation and approval are often implicit. In contrast, curriculum feedback is the least evident in the literature but appears in all our cases. Comparative work using functional equivalence can contribute to the literature by filling in gaps, but gaps in the literature make it more difficult to structure a range of comparable operations and therefore make deriving functional equivalents more difficult. In the worst case, biased literature may make it difficult to produce unbiased results – if the literature is entirely focused on how a certain operation works in one context, it may be difficult to identify a functional equivalent that captures the true range of all contexts. Moreover, it is difficult to find functional equivalents including operations that serve non-obvious functions that are not easy to observe. However, considering a broad range of literature to guide the identification of functions and functional equivalents can help researchers meet those challenges.

The aggregation level is not trivial for functionally equivalent operations (Grollmann, 2008). We observed different contexts drawing different boundaries around common problems. For example, some contexts differentiated career guidance and counselling from student enrolment and had separate operations for each – students identify the right path and then enrol. However, other contexts pool those two operations. Our analysis led us to pool those operations because so many contexts see them as one problem. This focus on equivalency helped us mediate between granular operations granularity in the literature and programme-level comparison. Overall, we only aggregated and refined the operations we found in the literature. We did not add any functions, nor did we significantly deviate from theory. This indicates that the literature provides a good foundation for starting this kind of analysis and that the analysis captures to what the literature is referring.

## Conclusion and outlook

This paper considers the value of a functional equivalence approach to comparative education. We extracted a concrete process – define the unit of analysis, use theory and literature to structure the range of comparable processes, empirically collect concrete operations related to those processes in the units under study, and decontextualise and deconstruct the operations to derive the common functions and identify functional equivalents. This provides a template future researchers can use in similar applications.

We apply the method to an exploration of the functional equivalents in similar education programmes across contexts. We show how a functional equivalence approach enables us to aggregate detailed information about the real-life operation of education programmes (shown in part C of the online supplemental material) into abstracted functions and functional equivalents that we can meaningfully compare. Our empirical analysis assesses how twelve different cases solve common problems, finding that every case has a solution for at least most problems and that their functionally equivalent solutions differ greatly in name, institutional setting, and the actors involved. An important advantage of the functional-equivalents approach is that it facilitates identification and comparison of equivalent functions despite these challenges.

We show that the functional equivalence approach reveals comparable processes across contexts, despite variation in socially constructed concepts and involved actors. The functions’ general similarity to the processes we identified in the literature shows that they also make sense theoretically. The diversity of actors involved in each function across cases demonstrates that the functional equivalence approach helps us abstract from specific contextual details and identify comparable units.

By nature, the functional equivalence approach abstracts away from the reality of education processes in context. Political, social, and economic realities may shape how these functions are achieved in practice. The purpose of identifying functional equivalents is to compare similar operations across countries. Comparative research and policy learning are often stymied by the different institutional and social characteristics of different contexts. The functional-equivalents approach is a method for meaningfully learning across contexts without resorting to a copy-paste approach.

We identify a few methodological challenges that future researchers should consider and address. For our analysis specifically, it was a challenge to identify the right experts to ask about functions in a programme. We used education leaders focused on the programmes under analysis and still found great diversity in the breadth and detail of their knowledge. While there are experts who can speak to operations in the whole CVC, some experts are knowledgeable about one programme part but not others. Experts give different levels of detail, necessitating multiple experts for one programme and aggregating responses before analysis. Finally, our application of the method did not focus on the development of programmes or operations over time. It would be possible to capture this element in a functional equivalence analysis, but that would require focussing on a single case over time rather than multiple cases.

We found two main challenges related to the use of functional equivalence as a method. First, this approach is not a shortcut that bypasses deep knowledge of the unit of analysis in each context under study. We relied on extensive case knowledge of each programme and our analysis may have been impossible without that knowledge. Second, the limitations of the literature related to the operations under study can make this kind of approach more difficult. A functional equivalence approach can certainly build the evidence and theory related to new areas, but weak theory makes it more difficult to structure the range of comparable operations and weak empirical work makes it more difficult to derive potential operations for coding. Biases in the literature – especially representation limited to certain contexts – can make it more difficult to abstract from concrete operations to functional equivalents that reflect the full range of practice.

The empirical evidence in this paper focuses on upper-secondary education programmes oriented toward labour market entry, and though those are the mainstream option for many countries, they can also be specific about the actors involved. Education programmes like these are especially diverse in the operations and actors involved since they must link education to employment. This set of functions provides a starting point for researchers who wish to compare this type of programme without becoming caught in the complexities of each context’s social, economic, and education systems.

This paper seeks to contribute to research on comparing education across contexts, which has always been complicated by the competing desires for comparability and contextuality. Our approach can serve as a starting point for future researchers who wish to compare education programmes and identify their functionally equivalent institutions across contexts (Rageth et al., 2021). As a caution, functionalism requires reducing complexity using abstract constructions of ‘comparative possibilities’ (Zolo, 1986: 118), which always limits generalizability. In respecting the challenges of cultural projection, institutional differences, and diverse solutions to the same problems, this approach is an important step forward for identifying comparable elements.

## Supplemental Material


Supplemental Material - Apples inside orange peels: Exploring the use of functional equivalents for comparing curriculum processes across contexts
Supplemental Material for Apples inside orange peels: Exploring the use of functional equivalents for comparing curriculum processes across contexts by Katherine M Caves, Ladina Rageth and Ursula Renold in Research in Comparative and International Education.

## Data Availability

The coded qualitative data that we analyse in this paper is provided in the online supplementary material.*

## Note

1.
https://uis.unesco.org/en/topic/international-standard-classification-education-isced/
