# Supplementary material for: Apples inside orange peels: Exploring the use of functional equivalents for comparing curriculum processes across contexts
Source: Res Comp Int Educ. 2024 Jun 12;19(3):261–80. doi: 10.1177/17454999241258928 (PMC11387131; doi:10.1177/17454999241258928)
Supplement: Supplemental Material - Apples inside orange peels: Exploring the use of functional equivalents for comparing curriculum processes across contexts [file sj-pdf-1-rci-10.1177_17454999241258928.pdf]

Apples inside orange peels: Identifying equivalent functions in education programmes

Online Supplemental Material

A Codebook

**Theory-driven codes**	**Process codes (in-vivo codes)**		
**Level 1:** Process phases	**Level 2:** Main processes	**Level 3:** Sub-level processes	**Level 4:** Responsible actors
			A Teachers
			B Companies
			C Professional associations
			D Ministry of Education (MoE)
			E Ministry of Labor
			F Policy makers
			G Pedagogical institute or teacher university
			H Researchers
			I Principals (Schools)
			J Ministry of Education affiliated agencies
			K Others

Theory-driven codes	Process codes (in-vivo codes)		
**Level 1:** Process phases	**Level 2:** Main processes	**Level 3:** Sub-level processes	**Level 4:** Responsible actors
**DESIGN phase**			
**1.00 Curriculum design**			
	1.10 Curriculum content development[1]		
		1.11 Method/process for curriculum development[2] (e.g., DACUM[3], Triplex-Method[4], Competence-oriented approach)	
		1.12 Curriculum content (job profiles, standards, goals, learning objectives, learning outcomes, skills, competences)	
		1.13 Curriculum framework[5]	
		1.14 Teaching and learning modes/methods (e.g., models of instruc-tion, concepts, procedures, dispositions)	
		1.15 Exam form (summative / formative)	
		1.16 Others	
	1.20 Curriculum consultation[6]		
		1.21 Consultation	
		1.22 Mitigation of	

1 Marsh and Willis ([1984] 1995), Marsh and Willis ([1984] 1995), Norton (1998), Billett (2006, 2011), Kelly ([1977] 2009), Rageth and Renold (2020)
2 Kelly ([1977] 2009)
3 Norton (1998)
4 Eggenberger, Rinawi, and Backes-Gellner (2018)
5 Marsh and Willis ([1984] 1995)
6 Vollstaedt (2003)

Theory-driven codes	Process codes (in-vivo codes)		
**Level 1:** Process phases	**Level 2:** Main processes	**Level 3:** Sub-level processes	**Level 4:** Responsible actors
		different opinions	
		1.23 Decision making act	
		1.24 Others	
	1.30 Curriculum approval[7]		
		1.31 Quality assurance	
		1.32 Approval by competent authority	
		1.33 Legal enactment	
		1.34 Submission for approval	
		1.35 Others	
**APPLICATION phase**			
**2.00 Curriculum application**			
	2.10 Career guidance and counselling[8]		
		2.11 Career guidance, counsellor preparation	
		2.12 Marketing material	
		2.13 Others	
	2.20 Student enrolment[9]		
		2.21 Matching process	
		2.22 Apprenticeship market	
		2.23 Access conditions	
		2.24 Others	
	2.30 Qualification of personnel[10]		
		2.31 Teachers’	

7 Finch and Crunkilton (1993)
8 Billett (2011)
9 Billett (2006, 2011)
10 Billett (2006, 2011), Tyler (2013)

Theory-driven codes	Process codes (in-vivo codes)		
**Level 1:** Process phases	**Level 2:** Main processes	**Level 3:** Sub-level processes	**Level 4:** Responsible actors
		continuing education	
		2.32 Instructors' continuing education	
		2.33 Exam experts' continuing education	
		2.34 Others	
	2.40 Syllabus, material, and infrastructure[11]		
		2.41 Syllabus for school	
		2.42 Syllabus for workplace/ on-the-job training	
		2.43 School learning material	
		2.44 Workplace/ On-the-job training material	
		2.45 School infrastructure	
		2.46 Workplace infrastructure	
		2.47 Others	
	2.50 Program delivery[12]		
		2.51 School teaching	
		2.52 Workplace training	
		2.53 Others	
	2.60 Assessment[13]	2.53 Conducting school exams	
		2.54 Conducting workplace exams	
		2.55 Certification	

[11] Finch and Crunkilton (1993), Marsh and Willis ([1984] 1995), Billett (2006, 2011), Rageth and Renold (2020)
[12] Finch and Crunkilton (1993), Billett (2011), Rageth and Renold (2020)
[13] Billett (2011), Kelly ([1977] 2009)

**Theory-driven codes**	**Process codes (in-vivo codes)**		
**Level 1:** Process phases	**Level 2:** Main processes	**Level 3:** Sub-level processes	**Level 4:** Responsible actors
		2.56 Quality assurance	
		2.57 Program output measurement	
		2.58 Others	
**FEEDBACK phase**			
**3.00 Curriculum feedback**			
	3.10 Information gathering[14]		
		3.11 Monitoring and evaluation framework	
		3.12 Research	
		3.13 Survey	
		3.14 Political intervention	
		3.15 Dissatisfied companies	
		3.16 Workshop	
		3.17 Others	
	3.20 Update initiation[15]		
		3.21 Regulation of time intervals (e.g., every 3-5 years)	
		3.22 Data beyond certain threshold	
		3.23 Proposal for update timing	
		3.24 Decision to update	
		3.25 Others	

[14] Finch and Crunkilton (1993), Billett (2011), Tyler (2013), Rageth and Renold (2020)
[15] Robinson (1981), Vollstaedt (2003), Kelly ([1977] 2009), Billett (2011), Tyler (2013), Rageth and Renold (2020)

B Questionnaire

Curriculum Processes

This survey refers to formal education and training programs which are formally regulated by the education authority of a country and have an education curriculum (sometimes also called education plan, standards, occupational standards, qualification standards, syllabus).

1. In your country or state, what is the biggest upper-secondary education program that prepares students to enter the job market?

Enter the program name, i.e. TVET, High School CTE, etc.

____

In the following questions, please refer your answers to **that program**.

2. At which level is this program regulated?

- ○ School level
- ○ District level
- ○ State Level
- ○ National level
- ○ I don't know

3. VET programs can have different curricula for the school part and the workplace learning parts. Please indicate which curriculum the program has, or which you mean when you answer the following questions on curriculum processes:

- ○ The program is mostly school-based, so the curriculum is for school.
- ○ The program has both school and workplace learning, and there is one curriculum that covers both parts
- ○ The program has both school and workplace learning, but I am answering only for the school part.
- ○ The program has both school and workplace learning, but I am answering only for the workplace learning part.

The following questions relate to different phases of the curriculum process. When answering these questions, please think about the program and curriculum you just selected. **program and curriculum you just selected**.

Explanations

- By <u>developing</u> the curriculum, we refer to any processes before defining the curriculum, any processes of defining the curriculum, and any processes of finalizing the curriculum.
- By <u>main processes</u>, we mean the main tasks that the involved parties and actors have to fulfill until the intended curriculum is developed.
- Please do not use any abbreviations and write the full names of processes and actors.

4. a) What are the <u>main processes</u> for <u>developing</u> the curriculum? Please list the 3-5 most important processes

**Process**	**Name**
**Before defining the curriculum**	
Process 1:*	[open text field]
Process 2:	[open text field]
Process 3:	[open text field]
**Defining the curriculum**	
Process 4:*	[open text field]
Process 5:	[open text field]
Process 6:	[open text field]
**Finalizing the curriculum**	
Process 7:*	[open text field]
Process 8:	[open text field]
Process 9:	[open text field]

**first process of each category is mandatory* (printed with leading asterisk: *first process of each category is mandatory)

b) Please give a short description for every process you entered in the previous question.

**Process**	**Short description**
**Before defining the curriculum**	
Process 1 from a.	[open text field]
Process 2 from a.	[open text field]
Process 3 from a.	[open text field]
**Defining the curriculum**	
Process 4 from a.	[open text field]
Process 5 from a.	[open text field]
Process 6 from a.	[open text field]

Finalizing the curriculum	
Process 7 from a.	[open text field]
Process 8 from a.	[open text field]
Process 9 from a.	[open text field]

c) Who is involved in each process? Please list the main actor groups for each process (e.g. teacher, business sector people):

Process	Involved actors
**Before defining the curriculum**	
Process 1 from a.	[open text field]
Process 2 from a.	[open text field]
Process 3 from a.	[open text field]
**Defining the curriculum**	
Process 4 from a.	[open text field]
Process 5 from a.	[open text field]
Process 6 from a.	[open text field]
**Finalizing the curriculum**	
Process 7 from a.	[open text field]
Process 8 from a.	[open text field]
Process 9 from a.	[open text field]

Curriculum Application

Explanations

- By applying the curriculum, we refer to the time before, during, and after program delivery.
- By main processes, we mean the main tasks that the involved parties and actors have to fulfill until the curriculum is delivered.
- Please do not use any abbreviations and write the full names of processes and actors.

5. **a) What are the main processes for applying the curriculum? Please list the 4-8 most important processes:**

Process	Name
**Before program delivery**	
1) [open text field]*	[open text field]
2) [open text field]	[open text field]
3) [open text field]	[open text field]
**During program delivery**	

4) [open text field]*	[open text field]
5) [open text field]	[open text field]
6) [open text field]	[open text field]
**End of program delivery**	
7) [open text field]*	[open text field]
8) [open text field]	[open text field]
9) [open text field]	[open text field]

b) Please give a short description for every process you entered in the previous question.

**Process**	**Short description**
**Before program delivery**	
Process 1 from a.	[open text field]
Process 2 from a.	[open text field]
Process 3 from a.	[open text field]
**During program delivery**	
Process 4 from a.	[open text field]
Process 5 from a.	[open text field]
Process 6 from a.	[open text field]
**End of program delivery**	
Process 7 from a.	[open text field]
Process 8 from a.	[open text field]
Process 9 from a.	[open text field]

c) Who is involved in each process? Please list the main actor groups for each process:

**Process**	**Involved actors**
**Before program delivery**	
Process 1 from a.	[open text field]
Process 2 from a.	[open text field]
Process 3 from a.	[open text field]
**During program delivery**	
Process 4 from a.	[open text field]
Process 5 from a.	[open text field]
Process 6 from a.	[open text field]
**End of program delivery**	
Process 7 from a.	[open text field]
Process 8 from a.	[open text field]
Process 9 from a.	[open text field]

Explanations

- By evaluating a curriculum, we mean any processes before and during the decision to revise the curriculum (after the decision, the curriculum development phase starts again).
- By main processes, we mean all tasks that the involved parties and actors have to fulfill until it is evaluated.
- Please do not use any abbreviations and write the full names of processes and actors.

6. a) What are the main processes for evaluating a curriculum? Please list the 4-8 most important processes:

Process	Name
**Before curriculum revision decision**	
1) Process 1:*	[open text field]
2) Process 2:	[open text field]
3) Process 3:	[open text field]
**During curriculum revision decision**	
4) Process 4:*	[open text field]
5) Process 5:	[open text field]
6) Process 6:	[open text field]

b) Please give a short description for every process you entered in the previous question.

Process	Short description
**Before curriculum revision decision**	
Process 1 from a.	[open text field]
Process 2 from a.	[open text field]
Process 3 from a.	[open text field]
**During curriculum revision decision**	
Process 4 from a.	[open text field]
Process 5 from a.	[open text field]
Process 6 from a.	[open text field]

c) Who is involved in each process? Please list the main actor groups for each process:

**Process**	**Involved actors**
**Before curriculum revision decision**	
Process 1 from a.	[open text field]
Process 2 from a.	[open text field]
Process 3 from a.	[open text field]
**During curriculum revision decision**	
Process 4 from a.	[open text field]
Process 5 from a.	[open text field]
Process 6 from a.	[open text field]

End of Survey

Thank you for participating in our survey.

7. **May we contact you again in a few months to discuss the results of the survey in a focus group?**
   - Yes
   - No

C Result tables for identifying functional equivalents

Table A1: Functional equivalent for curriculum content development (CD1)

Case number	1	2	3	4	5	6	7	8	9	10	11	12
**Global Region**	Africa	Europe	South America	Central America	Central America	North America	Asia	North America	North America	North America	Europe	Europe
**Economies by per capita GNI in 2012a**[16]	Low Income Country	Upper Middle Income country	High Income Country	Upper Middle Income country	Upper Middle Income country	High Income Country	Low Income Country	High Income Country	High Income Country	High Income Country	Upper Middle Income country	High Income Country
**Programme level**	National	National	National	National	National	District	National	School level	State Level	State Level	National	National
**Name of socially constructed operation(s)**	Committee to analyse skills needs	Qualification standard	Socialise with stakeholders; Sectorial learning objectives must be centred on labour and transversal skills of the sector, based on standards of competences and qualification	Qualification standard is identified	Determine occupational skills profile, leadership skills	Committee are formed to discuss curriculum development	Task analysis; Competency profiles; Curriculum development; focus groups	Define competencies and make reverse design of curriculum; are there workplace components	State standard, working with industry; Curriculum committee	Development of course sequences	Workshop with companies; Development of qualification	Development of qualification profile; Development of an education plan
**Actor groups involved in operation(s)**	D, C, H, K (Donors)	B, C, D	B, C, D, K (Stakeholders)	K (Vocational Institute)	A, K (experts)	D, B	D, J, A, C, F, K (experts, practitioners)	A, K (Industry experts)	A, B, K (academic colleague)	I, D, K (district agency)	C, J, G	C

Notes: The columns show all analysed cases and categorize them according to their global region, economy and programme level; For each programme, it shows the name of the socially constructed operations and the involved actor groups (A=teacher, B=company, C=professional association, D=Ministry of Education, E=Ministry of Labour, F=policy maker, G=pedagogical institute / teacher university, H=researcher, I=principal / school, J=Ministry of Education affiliated agency, K=Other.

[16] 2014wesp_country_classification.pdf

Table A2: *Functional Equivalence 2: Curriculum consultation (CD2, Code 1.20)*

**Case**	1	2	3	4	5	6	7	8	9	10	11	12
**Global region**	Africa	Europe	South America	Central America	Central America	North America	Asia	North America	North America	North America	Europe	Europe
**Economie by per capita GNI in 2012**	Low Income Country	Upper Middle Income Country	High Income Country	Upper Middle Income Country	Upper Middle Income Country	High Income Country	Low Income Country	High Income Country	High Income Country	High Income Country	Upper Middle Income Country	High Income Country
**Program**	National	National	National	National	National	District	National	School level	State Level	State Level	National	National
**Name of socially constructed process(es)**	-[17]	Preparing a draft of the curriculum; consultation among stakeholders; revision of document	Legitimation and validation with stakeholder; curriculum consultation	Conform, verify and validate the program; Plan evaluation	Expert consultation	-	Task verification	Once the curriculum is built; continue mapping to competencies and refine, and utilize industry for validation	Submit to curriculum committee; ensure that colleagues agrees with content	Review with school leadership and CTE Administrators; Validate with industry	-	Consultation among all relevant stakeholder groups
**Involved actor groups**		B, C (working group)	B, C, G, J, I, K (stakeholders)	K (Vocational Training Institute)	B, K (experts)	-	K (experts, facilitators)	A, K (experts)	K (academic colleagues)	B, C, D, J	-	C, D, K (states)

Notes: The columns show all analysed cases and categorize them according to their global region, economy and programme level; For each programme, it shows the name of the socially constructed processes and the involved actor groups (A=teacher, B=company, C=professional association, D=Ministry of Education, E=Ministry of Labour, F=policy maker, G=pedagogical institute / teacher university, H=researcher, I=principal / school, J=Ministry of Education affiliated agency, K=Other.

[17] If a case has no information about a specific process, this can either mean that this process does not exist in that case or that the expert who filled out the questionnaire was not sufficiently informed about it or did not think about this process.

Table A3: *Functional Equivalence 3: Curriculum approval (CD3, Code 1.30)*

**Case**	1	2	3	4	5	6	7	8	9	10	11	12
**Global region**	Africa	Europe	South America	Central America	Central America	North America	Asia	North America	North America	North America	Europe	Europe
**Economie by per capita GNI in 2012**	Low Income Country	Upper Middle Income Country	High Income Country	Upper Middle Income Country	Upper Middle Income Country	High Income Country	Low Income Country	High Income Country	High Income Country	High Income Country	Upper Middle Income Country	High Income Country
**Program**	National	National	National	National	National	District	National	School level	State Level	State Level	National	National
**Name of socially constructed process(es)**	After elaborating the curriculum, another committee has to validate the skills needs	Consistency check of recognizing authority; pedagogical assessment	Approbation or rejection by National Education Council	Evaluation of redesigned plan; approbation or rejection	Approval by ministry	Approval by local school boards	Curriculum is approved by the Council	Schools submit their CTE plans/curriculum authority for approval and oversight	Working with industry and faculty chairs; Industry advisory boards; ensure that content fits with overall curriculum; Check with chairperson	District office finalizes and approved the curriculum framework	Confirmation and adoption by the relevant bodies	Federal ministry of education approves
**Involved actor groups**	D, G, B, H, K	G	D	K	A, K (experts)	A, I, K (school board members)	F, D	D	A, B, I, J,	A, B, J.	C, D, K	D

Notes: The columns show all analysed cases and categorize them according to their global region, economy and programme level; For each programme, it shows the name of the socially constructed processes and the involved actor groups (A=teacher, B=company, C=professional association, D=Ministry of Education, E=Ministry of Labour, F=policy maker, G=pedagogical institute / teacher university, H=researcher, I=principal / school, J=Ministry of Education affiliated agency, K=Other.

Table A4: *Functional Equivalence 4: Student information and enrolment (CA1, codes 2.10 & 2.20)*

**Case**	1	2	3	4	5	6	7	8	9	10	11	12
**Global region**	Africa	Europe	South America	Central America	Central America	North America	Asia	North America	North America	North America	Europe	Europe
**Economie by per capita GNI in 2012**	Low Income Country	Upper Middle Income Country	High Income Country	Upper Middle Income Country	Upper Middle Income Country	High Income Country	Low Income Country	High Income Country	High Income Country	High Income Country	Upper Middle Income Country	High Income Country
**Program**	National	National	National	National	National	District	National	School level	State Level	State Level	National	National
**Name of socially constructed process(es)**	-	Career Guidance Material; Student enrollment	-	The information session for students is organized; Students are selected and enrolled	A process is carried out from the primary level with emphasis on the secondary in the 9th grade so that the student chooses a career related to his interest.	-	-	Target enrollment strategy; market the program for students	-	-	Contracting companies for Work-based learning; schools and companies cooperate in the enrolment process.	-
**Involved actor groups**	-	A	-	K (vocational training institute)	A, K (experts)	-	-	A, B, K (administration)	-	-	B, I	-

Notes: The columns show all analysed cases and categorize them according to their global region, economy and programme level; For each programme, it shows the name of the socially constructed processes and the involved actor groups (A=teacher, B=company, C=professional association, D=Ministry of Education, E=Ministry of Labour, F=policy maker, G=pedagogical institute / teacher university, H=researcher, I=principal / school, J=Ministry of Education affiliated agency, K=Other.

Table A5: *Functional Equivalence 5: Qualification of personnel (CA2, code 2.30)*

**Case**	1	2	3	4	5	6	7	8	9	10	11	12
**Global region**	Africa	Europe	South America	Central America	Central America	North America	Asia	North America	North America	North America	Europe	Europe
**Economie by per capita GNI in 2012a**[18]	Low Income Country	Upper Middle Income Country	High Income Country	Upper Middle Income Country	Upper Middle Income Country	High Income Country	Low Income Country	High Income Country	High Income Country	High Income Country	Upper Middle Income Country	High Income Country
**Program**	National	National	National	National	National	District	National	School level	State Level	State Level	National	National
**Name of socially constructed process(es)**	Govt. selects public TVET institutions and prepares them for curriculum implementati on	Train teachers; Train Professionals	Define internal capabilities for curriculum adoption and gaps and take strategic actions	The VET Center is determined; availability of resources and instructors Is organized; instructors are assigned	Training for teachers and specialists (also for business staff); methodologica l preparation for teachers and specialists	-	Capacity building of instructors	Hire appropriate staff/faculty	-	Professional development to teachers	-	-
**Involved actor groups**	D,E, K	G, I (train the trainer)	I, A	K (Vocational training institute)	A, K (methodologist s)	A, I	J, A, C	B, K (administratio n)		J, A, K (guidance counselor)	-	-

Notes: The columns show all analysed cases and categorize them according to their global region, economy and programme level; For each programme, it shows the name of the socially constructed processes and the involved actor groups (A=teacher, B=company, C=professional association, D=Ministry of Education, E=Ministry of Labour, F=policy maker, G=pedagogical institute / teacher university, H=researcher, I=principal / school, J=Ministry of Education affiliated agency, K=Other.

[18] 2014wesp_country_classification.pdf

Table A6: *Functional Equivalence 6: Resource provision (CA3; code 2.40)*

**Case**	1	2	3	4	5	6	7	8	9	10	11	12
**Global region**	Africa	Europe	South America	Central America	Central America	North America	Asia	North America	North America	North America	Europe	Europe
**Economie by per capita GNI in 2012**	Low Income Country	Upper Middle Income Country	High Income Country	Upper Middle Income Country	Upper Middle Income Country	High Income Country	Low Income Country	High Income Country	High Income Country	High Income Country	Upper Middle Income Country	High Income Country
**Program**	National	National	National	National	National	District	National	School level	State Level	State Level	National	National
**Name of socially constructed process(es)**	-	Preparing teacher material; execution & assuring infrastructure	Review and school based analysis; internal strategic planning: course planning, teaching teams, schedule; learning tools	The teaching profile is analysed; the curriculum or course listing of the plan is determined; instructors are assigned. Tools and materials are acquired	Elaboration of the curriculum (subjects to be shared in each year of study are determined by methodologis ts and experts); elaboration of the subject programs; Define material resources to fulfill the program	Courses and content standards are approved, and teachers are involved in curriculum mapping	-	Identify instructional delivery plans, work with businesses to shore up work based learning components	Individual schools decide how to implement the curriculum; Educators develop lesson plans;	Teachers develop lesson plans	Development of plan of work-based learning	National, regional or school-specific curricula will be developed
**Involved actor groups**	-	A, B, C, G, I	A, I	K (career leaders, curriculum developers; vocational training institute)	A, K (experts)	A, K (supervisors)	-	A, B, K	A	A	A, B, C	A, C

Notes: The columns show all analysed cases and categorize them according to their global region, economy and programme level; For each programme, it shows the name of the socially constructed processes and the involved actor groups (A=teacher, B=company, C=professional association, D=Ministry of Education, E=Ministry of Labour, F=policy maker, G=pedagogical institute / teacher university, H=researcher, I=principal / school, J=Ministry of Education affiliated agency, K=Other.

Table A7: Functional Equivalence 7: Program delivery (CA4; code 2.50)

Case	1	2	3	4	5	6	7	8	9	10	11	12
**Global region**	Africa	Europe	South America	Central America	Central America	North America	Asia	North America	North America	North America	Europe	Europe
**Economie by per capita GNI in 2012**	Low Income Country	Upper Middle Income Country	High Income Country	Upper Middle Income Country	Upper Middle Income Country	High Income Country	Low Income Country	High Income Country	High Income Country	High Income Country	Upper Middle Income Country	High Income Country
**Program**	National	National	National	National	National	District	National	School level	State Level	State Level	National	National
**Name of socially constructed process(es)**	For the implementati on of the program, a regular monitoring has to be carried out. This strategy aims to ensure the quality of the training and to identify the barriers faced in the implementati on process.	Execution & Assuring Infrastructure	Planning the year of each course; learning structure; Planning each lesson	The program is executed and evaluated. It is the execution of each course, carrying out evaluation actions at the end of each course. The certificate is awarded	Vocational training process; define resources to fulfill the program, resources needed for skill development and goal achievement are made available	Teachers deliver instruction and provide opportunities for collaborative, inquiry based learning.	Instructors deliver the curriculum in schools; instructor assess the learning of students at school and mostly internal assessment; final examination is done by CTEVT[19]	Project-based learning; experimental learning; assess if students met all objectives and competences	Educators tweak lesson plans as they implement; teacher delivery and assessments;	Teacher delivers content; ongoing professional development and resources which help to improve implementati on	Implementati on, realization of all classes with 3 or 4 year profiles;	Delivery curriculum for workplace, school and intercompany courses
**Involved actor groups**	D, G.	A, B, C, I	A, I	K (vocational training institute)	A, K (experts)	A, K	A, K (administrator s)	A	A	A, J	A, B, K	B, C

Notes: The columns show all analysed cases and categorize them according to their global region, economy and programme level; For each programme, it shows the name of the socially constructed processes and the involved actor groups (A=teacher, B=company, C=professional association, D=Ministry of Education, E=Ministry of Labour, F=policy maker, G=pedagogical institute / teacher university, H=researcher, I=principal / school, J=Ministry of Education affiliated agency, K=Other.

---

19 CTEVT = Council for Technical Education and Vocational Training

Table A8: *Functional equivalent for programme assessment and certification (CA5)*

**Case number**	1	2	3	4	5	6	7	8	9	10	11	12
**Continent**	Africa	Europe	South America	Central America	Central America	North America	Asia	North America	North America	North America	Europe	Europe
**Economies by per capita GNI in 2012a**[20]	Low Income Country	Upper Middle Income country	High Income Country	Upper Middle Income country	Upper Middle Income country	High Income Country	Low Income Country	High Income Country	High Income Country	High Income Country	Upper Middle Income country	High Income Country
**Programme level**	National	National	National	National	National	District	National	School level	State Level	State Level	National	National
**Name of socially constructed operation(s)**	-	Organizing testing, examination; output-check	Standardized tests; Centralized design and application; Feedback loop between quality agency and schools; internal analysis and adjustments	Evaluation of the results with a view to excellence and quality	The evaluation system is determined by the scope of the subject	Student outcomes are reviewed and modifications to curriculum and instructor will occur as necessary	Assessment of effectiveness of programme in terms of certification, employment rate; final examination; tracer studies, employer satisfaction survey; Quality Assurance monitoring	Solicit feedback, make changes when needed (continuous improvements); ensure student progress and success; engage industry to gauge effectiveness of work based learning; where students qualified for the job market?	Feedback and validation of content; making sure there is quality control; assess outcomes	Monitoring, reporting and technical assistance	Final exam	-
**Actor groups involved in operation(s)**	-	A, B, I	A, D, K (agency)	K (career leaders)	K (methodologists)	A, K	J, K (examination of controller)	A, B, K (administration)	A, B	A, J	A, B	-

Notes: The columns show all analysed cases and categorize them according to their global region, economy and programme level; For each programme, it shows the name of the socially constructed operations and the involved actor groups (A=teacher, B=company, C=professional association, D=Ministry of Education, E=Ministry of Labour, F=policy maker, G=pedagogical institute / teacher university, H=researcher, I=principal / school, J=Ministry of Education affiliated agency, K=Other

[20] 2014wesp_country_classification.pdf

Table A9: *Functional Equivalence 9: Information gathering (CF1, code 3.10)*

**Case**	1	2	3	4	5	6	7	8	9	10	11	12
**Global region**	Africa	Europe	South America	Central America	Central America	North America	Asia	North America	North America	North America	Europe	Europe
**Economie by per capita GNI in 2012**	Low Income Country	Upper Middle Income Country	High Income Country	Upper Middle Income Country	Upper Middle Income Country	High Income Country	Low Income Country	High Income Country	High Income Country	High Income Country	Upper Middle Income Country	High Income Country
**Program**	National	National	National	National	National	District	National	School level	State Level	State Level	National	National
**Name of socially constructed process(es)**	Impact evaluation studies have been conducted; Public and private institutions have to collaborate to update the curriculum	Evaluation (Monitoring); collecting data and information about changes	Perform annual monitoring of trajectory results	Check if the qualification standard has to be modified; Relevance and validity of the plans is analyzed, based on the market and global trends; Assessment of required improvement s	Validity and current relevance of the content	Program outcomes are reviewed by teachers, administrators and other stakeholders; Data regarding post-secondary standards, and in some cases employability standards is considered	Collect feedback from employers, training schools, teachers, instructors	Identify the need; Examine regional market with industry; Identify best practices in the field if other regions have the program	Labor market data analysis to contemplate the new program; Survey; Looking at competitor programs; Gathering feedback; Student outcomes	Labor market analysis; Industry interview	Workshop with companies to evaluate job profiles; Development of a new curriculum	Review of the professional profiles; Workshop about curriculum update;
**Involved actor groups**	D, K (Ministry of Plan)	C, B, H, K (external institution)	A, D	A, G, K (Vocational Training Institute, experts)	A, K (experts)	A, K (administratio n, stakeholders)	B, A, K (CTEVT Curriculum division)	H, K (experts, economists)	H, B, A	A, B, D, H, J	B, C, J	C,D, K (cantons)

Notes: The columns show all analysed cases and categorize them according to their global region, economy and programme level; For each programme, it shows the name of the socially constructed processes and the involved actor groups (A=teacher, B=company, C=professional association, D=Ministry of Education, E=Ministry of Labour, F=policy maker, G=pedagogical institute / teacher university, H=researcher, I=principal / school, J=Ministry of Education affiliated agency, K=Other.

Table A10: *Functional Equivalence 10: Update initiation (CF2, code 3.20)*

**Case**	1	2	3	4	5	6	7	8	9	10	11	12
**Global region**	Africa	Europe	South America	Central America	Central America	North America	Asia	North America	North America	North America	Europe	Europe
**Economie by per capita GNI in 2012**	Low Income Country	Upper Middle Income Country	High Income Country	Upper Middle Income Country	Upper Middle Income Country	High Income Country	Low Income Country	High Income Country	High Income Country	High Income Country	Upper Middle Income Country	High Income Country
**Program**	National	National	National	National	National	District	National	School level	State Level	State Level	National	National
**Name of socially constructed process(es)**	The Government has to evaluate the implementation of the program; Committee to analyse the skills needs.	Identify up-dating time; Decide how often curricula will need to be changed; Define relevant stakeholders to be involved	-	Program update, if necessary with the formation of the technical team	The commission of specialists defines what contents have changed and are no longer implemented; update of productive processes	Major changes must be approved by local school boards	Curriculum division collects the required information for revision and recommend to CTEVT for the revision; CTEVT decides, revision is done every 3-5 years in time	Refine curriculum for next cohort; Ensure faculty and industry advisory board are in the loop; Faculty and industry advisory board work together to ensure revisions meet the benchmarks	Curriculum and instructional design; Impact on other aspects of the curriculum; Review overall goals	-	Confirmation and adoption by the relevant bodies	curriculum update
**Involved actor groups**	D, K (ministry of plan)	B, C, D	-	K (vocational training center)	A, K (experts)	A, K	A, B, F, D, J, K (curriculum division)	A, B, K	A, B	-	D	-

Notes: The columns show all analysed cases and categorize them according to their global region, economy and programme level; For each programme, it shows the name of the socially constructed processes and the involved actor groups (A=teacher, B=company, C=professional association, D=Ministry of Education, E=Ministry of Labour, F=policy maker, G=pedagogical institute / teacher university, H=researcher, I=principal / school, J=Ministry of Education affiliated agency, K=Other.

D References

Billett, Stephen. 2006. "Constituting the Workplace Curriculum." *Journal of Curriculum Studies* 38 (1):31-48. 10.1080/00220270500153781.

———. 2011. *Vocational Education: Purposes, Traditions and Prospects*. Dordrecht, DE: Springer.

Eggenberger, Christian, Miriam Rinawi, and Uschi Backes-Gellner. 2018. "Occupational specificity: A new measurement based on training curricula and its effect on labor market outcomes." *Labour Economics* 51:97-107. 10.1016/j.labeco.2017.11.010.

Finch, Curtis R., and John R. Crunkilton. 1993. *Curriculum Development in Vocational and Technical Education. Planning, Content, and Implementation*. 4th ed. Boston, US: Allyn and Bacon.

Kelly, Albert Victor. [1977] 2009. *The Curriculum: Theory and Practice*. 6th ed. Thousand Oaks, CA: Sage.

Marsh, Colin J., and George Willis. [1984] 1995. *Curriculum: Alternative Approaches, Ongoing Issues*. 3rd ed. Englewood Cliffs, NJ: Merrill.

Norton, Robert E. 1998. "Quality Instruction for the High Performance Workplace: DACUM." *ERIC Document Reproduction Service No. ED 419 15*. https://files.eric.ed.gov/fulltext/ED419155.pdf.

Rageth, Ladina, and Ursula Renold. 2020. "The Linkage Between the Education and Employment Systems: Ideal Types of Vocational Education and Training Programs." *Journal of Education Policy* 35 (4):503-28. 10.1080/02680939.2019.1605541.

Robinson, S. B. 1981. *Bildungsreform als Revision des Curriculum. Ein Strukturkonzept für Curriculumentwicklung, Arbeitsmittel für Studium und Unterricht*. Neuwied/Darmstadt, DE: Luchterchand.

Tyler, Ralph W. 2013. *Basic principles of curriculum and instruction*. Chicago and London: University of Chicago Press.

Vollstaedt, Witlof. 2003. "Steuerung von Schulentwicklung und Unterrichtsqualität durch staatliche Lehrpläne? ." In *Recht - Erziehung - Staat (Zeitschrift für Pädagogik, 47. Beiheft)*, edited by Hans-Peter Fuessel and Peter M. Roeder, 194-214. Weinheim, Basel, Berlin: Beltz Verlag.
